# Erythropoietin Pretreatment Effect on Blood Glucose and Its Relationship With Inflammatory Factors After Brain Ischemic-Reperfusion Injury in Rats

**DOI:** 10.32598/bcn.9.5.347

**Published:** 2018-09-01

**Authors:** Raheleh Gholamzadeh, Mehdi Eskandari, Mohammad Reza Bigdeli, Hossein Mostafavi

**Affiliations:** 1. Department of Physiology and Pharmacology, School of Medicine, Zanjan University of Medical Sciences, Zanjan, Iran.; 2. Department of Animal Science, Faculty of Biological Sciences, Shahid Beheshti University, Tehran, Iran.

**Keywords:** Erythropoietin, Pretreatment, Blood glucose, Inflammatory factors, Brain ischemia

## Abstract

**Introduction:**

Brain Ichemic-Reperfusion Injury (IRI) activates different pathophysiological processes. It also changes physiological parameters such as Blood Glucose (BG) level. An increase in BG after stroke is associated with poor clinical outcomes. Erythropoietin has been shown to be effective on both reducing inflammation and BG level. Therefore, in this study the erythropoietin pretreatment effect on BG and its relationship with inflammatory markers after brain IRI was investigated.

**Methods:**

Thirty adult male Wistar rats were randomly divided into 5 groups: sham, control and 3 pretreatment groups: single dose, double dose, and triple dose that received 1000 U/kg of erythropoietin before stroke induction in different times intraperitoneally. A rat model of IRI was established by Middle Cerebral Artery Occlusion (MCAO) for 60 minutes. Infarct volume, neurological defects, Interleukin-1α (IL-1α) and IL-6 serum levels were evaluated 24 hours after reperfusion. Also BG was measured after 1, 6, and 24 hours.

**Results:**

Single dose of erythropoietin significantly decreased infarct volume and improved neurological defects which was associated with decreased serum level of IL-1α and IL-6 but higher doses of erythropoietin administration had adverse effects on histological, neurological, and inflammatory results. In addition, erythropoietin significantly increased BG in a dose-dependent manner.

**Conclusion:**

Erythropoietin could reduce brain IRI by reducing inflammation and BG stabilization. The results of the present study demonstrated a relationship between inflammatory factors and hyperglycemia after IRI and suggested that erythropoietin may be useful for preventing brain IRI, but its higher doses should be used with caution due to possible side effects.

## Highlights

- Brain ischemia increases blood glucose, inflammatory factors, and neurological deficits.
- There is a relationship between increment of blood glucose, inflammation, and clinical outcomes.
- Erythropoietin pretreatment improves outcome after brain ischemia by decreasing inflammation and blood glucose stabilization.
- Administration of erythropoietin in high dose has side effects and should be used with caution.

## Plain Language Summary

Stroke is one of the most important causes of mortality in the world. Many conditions, for example heart bypass operation, transplantation, sickle cell anemia, and so on are risk factors for stroke. After the stroke, the physiological functions of the body, such as blood pressure, blood sugar, body temperature, cell metabolism, oxygen saturation, etc., are impaired and in this situation different pathological process are activated. These physiological changes have important effects on increasing stroke damage, which are often neglected in experimental studies. Attention to association of physiological and pathological agents have clinical importance in the prevention of stroke. Therefore, in this study, we examined the erythropoietin pretreatment effect on blood glucose, inflammatory factors and its association with stroke volume and clinical outcomes. The results of our study showed that administration of erythropoietin protects the brain against stroke, but this drug should be used with caution in high-risk individuals because of its side effects and possibility of stroke volume increase.

## 1. Introduction

Ischemic stroke is the leading cause of death in the worldwide that imposes high costs on individuals and society (Yuen et al., 2011). Heart bypass surgery, transplantation, sickle cell anemia and heart attack are risk factors for cerebral ischemia (Bigdeli & Mohagheghi, 2014). Despite its enormous advances, medical science still lacks a safe and effective treatment for stroke (Yip et al., 2011). Thrombolytic therapy is the only safe and effective method for acute management of ischemic stroke which has a golden time and usage limitation (Yuen et al., 2011). Recently, preconditioning mechanisms, like pharmacological preconditioning are used to increase tolerance to ischemia (Gholamzadeh, Eskandari, Mostafavi, & Bigdeli, 2016).

Cerebral ischemia leads to the activation of inflammatory processes, and different metabolic and electrophysiological disorders (Bigdeli & Mohagheghi, 2014; Shah, 2000). Reperfusion after ischemic injury is associated with a greater harm, i.e. Ischemic-Reperfusion Injury (IRI) (Bigdeli & Mohagheghi, 2014; Paschos, Lykissas, & Beris, 2008; Wu et al., 2014). In addition, cerebral ischemia changes body physiological conditions such as blood oxygen saturation, blood sugar, temperature and blood pressure (Radermecker & Scheen, 2010). Hyperglycemia is common in the early stroke phase and has been observed in two-thirds of all types of ischemic stroke (Lindsberg & Roine, 2004). Studies demonstrated that hyperglycemia is associated with worse outcomes, enhanced infarct volume and increased risk for post-stroke mortality (Bhalla, Wolfe, & Rudd, 2001; Lindsberg & Roine, 2004; Mehta, 2003). Oxidative glucose metabolism is perturbed following ischemia and causes lactic acidosis and vasogenic edema (Bhalla et al., 2001).

Hyperglycemia increases the production of lactic acid, leading to neuronal damage (Bhalla et al., 2001; Mehta, 2003). Also, both hyperglycemia and hypoglycemia lead to an increased production of free radicals and inflammatory factors such as Interleukin-1 (IL-1) and IL-6 (Shukla, Shakya, Perez-Pinzon, & Dave, 2017). A neuroendocrine stress and inflammatory response may contribute to increased blood glucose after a stroke. Thus, maintaining physiological homeostasis and reducing inflammation after a stroke has an important role in therapeutic achievements and mortality reduction (Wong & Read, 2008).

Erythropoietin is a glycoprotein hormone. It is secreted by the liver in fetal and synthesized in the kidney in adults (Chen et al., 2015; Wu et al., 2014). Erythropoietin was initially applied to treat anemia (Yao et al., 2016). The protective effect of erythropoietin preconditioning on reducing IRI and inflammatory responses has been investigated in the brain (Yu, Fan, Sun, Yao, & Chai, 2016), heart (Rong & Xijun, 2015), kidney (Elshiekh, Kadkhodaee, Seifi, Ranjbaran, & Ahghari, 2015; Liao, Li, Wang, & Xie, 2016), intestine (Kai-Lan & Si, 2015), liver (Liu et al., 2015), and lung (Wu et al., 2006). Erythropoietin could reduce infarct size and improve neurological deficits (Yuen et al., 2011).

Several studies have determined the effects of erythropoietin on decreased blood glucose and an increased glucose tolerance in diabetic rats (Chen et al., 2015; Meng, Zhu, Bi, Yang, & Wang, 2013; Niu, Chang, Niu, Cheng, & Lee, 2016). To our knowledge, no study has explored the effects of erythropoietin pretreatment on blood glucose in non-diabetic stroke patients and its relationship with inflammatory factors and outcomes. Therefore the present study investigated the impact of erythropoietin on improved IRI by reducing blood glucose and inflammatory factors.

## 2. Methods

### 2.1. Experimental groups

**Figure 1.**
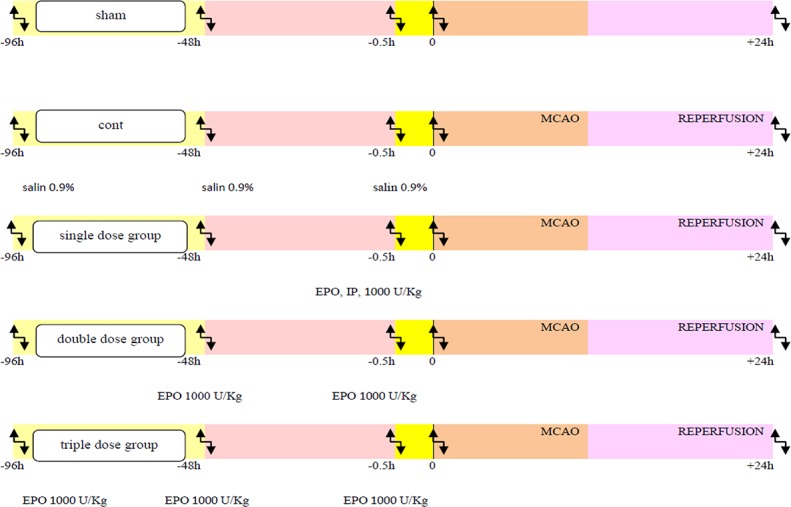
Schematic representation of the experimental groups Stroke induced by middle cerebral artery occlusion model and blood flow was established after 1 hour. Tissue and blood parameters were assessed 24 hours after the reperfusion.

According to Figure 1, 30 adult male Wistar rats (weighing 200 to 300 g) were maintained under standard conditions (12:12 h light-dark cycle and controlled temperature of 24±2°C). Then, the rats were ran-domly divided into 5 groups (6 rat in each group): sham (receiving surgical stress), control (Middle Cerebral Artery Occlusion [MCAO] model receiving saline 0.9%) and 3 pretreatment groups: single dose pre-treatment group (30 minutes before the stroke induction), double dose (0.5 and 48 hours before), triple dose (0.5, 48 and 96 hours before the stroke induction) that received 1000 U/kg of recombinant human erythropoietin alpha (ampoule Eprex 4000 unit, code:1228048326 prepared by Pooyesh Darou Pharmaceuticals company) intraperitoneally before the stroke induction (Figure 1).

The animals were anesthetized 30 minutes after the last injection by chloral hydrate (Merck, Germany, Code: 102425). Brain ischemia was induced by MCAO model for 60 minutes. Then, the animals were evaluated in terms of stroke volume, blood glucose, inflammatory factors (IL1-α & IL6) and motor neurological deficits 24 hours after the stroke induction.

### 2.2. Induction of focal cerebral ischemia by MCAO model

To close the Middle Cerebral Artery (MCA) and induction of ischemia, the weighed animals were anesthetized with chloral hydrate (400 mg/kg) and placed in the supine position on the surgical pad. Under cardiac monitoring, MCAO surgery model was performed in accordance with Longa et al. method. The surgical site was sterilized with 70% alcohol. A linear 2-cm incision was made on the right side of the neck along the spine of the animal. Under a microscopic surgery by pushing the salivary tissue and sternohyoid muscle, common carotid artery appeared which was separated from the vagus nerve. Then, a silicone coated size 3-0 inch nylon suture with a round tip was entered to the external carotid artery and pushed through internal carotid artery to reach the MCA. A slight resistance against suture after passing 20–22 mm of suture length indicated that it is placed in the correct location. Blood flow was restored after 60 minutes. During the surgery, rectal body temperature was measured and maintained at about 37°C (Bigdeli, 2008; Chiang, Messing, & Chou, 2011).

### 2.3. Measurement of stroke volume

**Figure 2.**
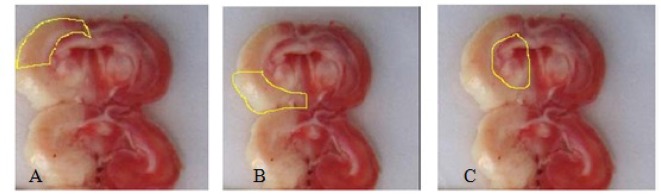
Separation of penumbra (A), core (B) and subcortex (c) by Image-J Software

The animals were sacrificed by chloral hydrate (800 mg/kg) and decapitated 24 hours after induction of ischemia, to measure infarct volume. Then, their brain was removed immediately and placed in cool normal saline (4°C) for 10 minutes. Seven brain coronal sections were cut at 2 mm in thickness from frontal to temporal lobes, according to rat brain matrix. Tissue sections were immersed in triphenyltetrazolium chloride (Sigma, USA, code: 1.38380) solution and incubated at 37°C for 10 minutes. Then, images were taken from sections using a digital camera and transferred to a computer for analysis by Image-J Software. As shown in Figure 2, stroke volume was calculated according to the following formula in core, penumbra, and subcortex regions (Bigdeli, 2008; Yuen et al., 2011).

$$\text{Brain}\;\text{infarct}\;\text{volume}\;=\frac{\text{Left}\;\text{hemisphere}\;\text{volume}-\left(\text{Right}\;\text{hemisphere}\;\text{volume}-\text{Infarct}\;\text{volume}\right)}{\text{Left}\;\text{hemisphere}\;\text{volume}}$$

### 2.4. Measurement of blood glucose

Blood glucose levels were controlled at 1, 6 and 24 hours after the stroke induction by a glucometer (Accu-Chek Performa) using the animals’ tail vein blood samples (Chen et al., 2015).

### 2.5. Inflammatory factors of interleukin-1α and interleukin-6

Two milliliters of the rats’ blood samples were taken to measure IL-1α and IL-6, 24 hours after the induction of stroke. Blood samples were transferred to sterile tubes and centrifuged at 4000 rpm, at 4°C. The serum samples were stored at −70°C in microtubes. Serum levels of IL-1α and IL-6 were measured by ELISA method according to Dia-clone France protocol kit (Gul, Yasim, & Aral, 2009).

### 2.6. Assessment of motor neurological deficits

Animals’ neurological defects were evaluated by standard Bederson criteria. The animals’ neurological status were scored 24 hours after the induction of ischemia. The interpretation of scores is as follows:

Score (0): No neurological deficits were observed; Score (1): Complete failure on the anterior surface of the front paws (mild focal neurological deficits); Score (2): Turning to the left (moderate focal neurological deficits); Score (3): Falling to the left (severe focal neurological deficits); Score (4): Animal is incapable of walking spontaneously and has a low level of consciousness; and Score (5): Death (Bigdeli, 2008).

### 2.7. Statistical analysis

The collected results were analyzed by SPSS using one-way Analysis of Variances (ANOVA) and LSD post-hoc analysis. Also, neurological deficits were analyzed by Mann–Whitney U test. The obtained results are expressed as Mean±SEM and P<0.05 is considered as statistically significant.

## 3. Results

### 3.1. Stroke volume and motor neurological deficits

**Figure 3.**
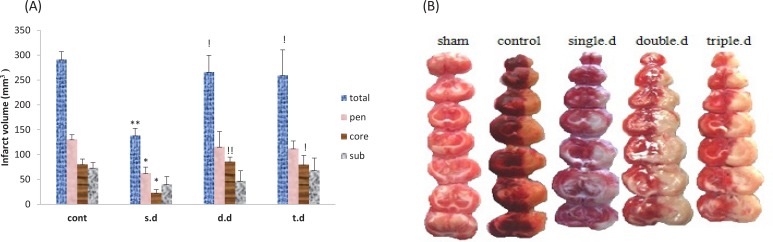
Comparison of erythropoietin pretreatment effect (1000 U/kg) on tissue damage volume in the treatment groups A: Single dose (s.d); Double dose (d.d); Triple dose (t.d) with control group in penumbra, core, subcortex and total volume. ^*^P<0.05 and ^**^P<0.01 are significant differences compared to the controls. B: Sections of brain tissue in different groups were stained with the characteristics of red in safe areas and white in the affected areas. ^!^P<0.05 are ^!!^P<0.007 are significant differences with single dose group.

A single dose of erythropoietin pretreatment significantly reduced total stroke volume and stroke volume of core and penumbra regions. Also it improved neurological deficit scores compared to the control group (P<0.05). Double and triple doses of erythropoietin pretreatment had no effect on stroke volume and neurological deficit scores. Moreover, significant differences were observed between pretreatment groups with regard to infarct volume and neurological deficits (P<0.05). (Figure 3 and Table 1)

**Table 1.** Comparing neurological deficits between the control and experimental groups according to Bederson criteria

**Groups**	**Median**	**Mean**	**Neurological Deficits Grading**						**Total**	**Sig.**

			**0**	**1**	**2**	**3**	**4**	**5**		
Control	4	4.066	0	0	2	3	2	8	15	
Single dose	3	2.866	1	1	5	2	3	3	15	P<0.05^*^
Double dose	4	4.266	0	0	2	0	5	8	15	P<0.05^#^
Triple dose	4	3.666	0	1	3	2	3	6	15	

* P<0.05 is the significant differences with the control group and

# P<0.05 is the significant differences with double dose group.

### 3.2. Blood glucose

**Figure 4.**
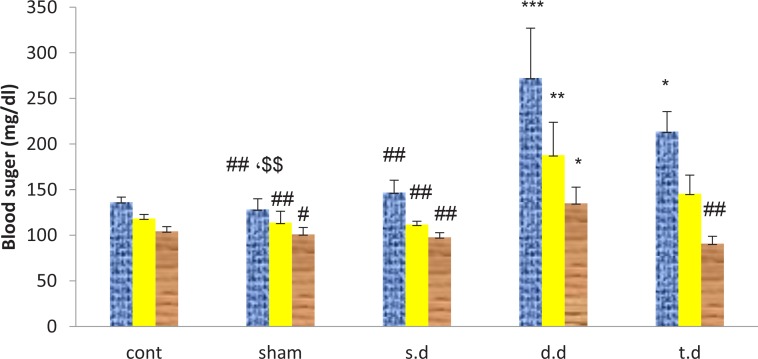
Blood glucose at 1, 6 and 24 hours after stroke induction in the sham, control and experimental groups ^*^P<0.05, ^**^P=0.003, ^***^P<0.000: significant differences with the controls; ^#^ P<0.05: significant differences with the double dose group; ^##$$^P<0.01: significant differences with the double dose (#) and triple dose ($) groups.

The blood glucose was measured at 1, 6 and 24 hours after brain ischemia in all groups which it had no significant difference in control group compared to the sham. Erythropoietin pretreatment increased blood glucose after stroke. This enhancement was significant in the double and triple dose erythropoietin pretreatment groups, compared to control and single dose groups; especially in the first and sixth hours after the stroke (P<0.05). Also, all experimental groups showed a reduction in blood glucose within 24 hours after the stroke (Figure 4).

### 3.3. Inflammatory factors: Interleukin-1α and interleukin-6

**Figure 5.**
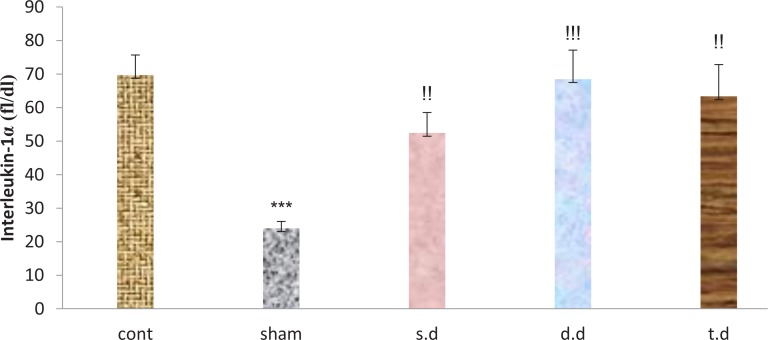
Serum levels of interleukin-1α 24 hours after stroke induction in the sham, control, and experimental groups ^***^P<0.000 indicates significant differences, compared to the controls; ^!!^P<0.01 and ^!!!^P<0.000 indicate significant differences, compared to the sham group.

**Figure 6.**
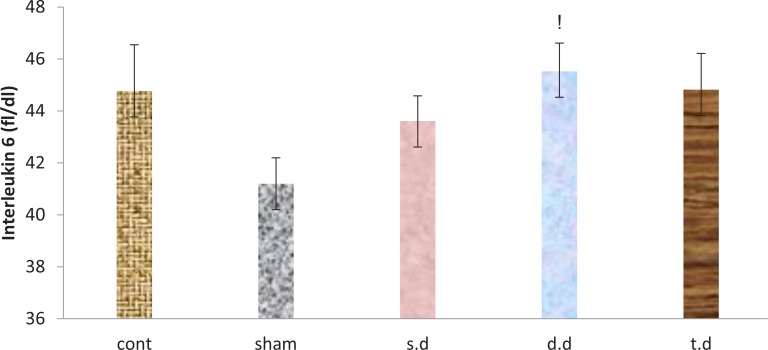
Serum levels of interleukin-6 24 hours after stroke induction in the sham, control and experimental groups ^!^P<0.05 indicates significant differences compared to the sham group.

Serum inflammatory factors interleukin-1-α and interleukin-6 increased after stroke in all groups, compared to the sham group in which this rise was significant in IL-1α. A single dose of erythropoietin pretreatment reduced serum levels of IL-1α and IL-6, compared to the control group. However, this reduction was not significant. Double and triple dose erythropoietin pretreatment had no effect on IL-1α and IL-6 serum levels among the groups (Figures 5 and 6).

## 4. Discussion

Blood flow maintenance in the ischemic area is among the main objectives in stroke treatment. However, restoration of blood flow in the damaged area may aggravate the injury. In addition, post-stroke changes in different physiological conditions of the body such as blood glucose, blood saturation, and temperature could increase the severity of the lesion. Therefore, it is important to prevent future damage after reperfusion. Stabilizing physiological conditions and reducing levels of inflammation can decrease the injury caused by the stroke and reperfusion (Gentile, Seftchick, Huynh, Kruus, & Gaughan, 2006; Wong & Read, 2008; Yu et al., 2016).

A study demonstrated that erythropoietin pretreatment has a positive effect on the infarct volume and neurological deficits (Yu et al., 2016). The results of our study suggested that injecting a single dose of erythropoietin pretreatment (1000 U/kg) 30 minutes before the stroke induction, significantly reduced infarct volume damage and improved neurological deficits. These results are consistent with results of previous studies. However, the results regarding higher doses of erythropoietin were inconsistent with the previous experimental studies. These diverse results may be due to different time and dosage of erythropoietin administration.

We recognized that pretreatment with a single dose of erythropoietin can also positively affect IRI. In contrast with the prior research, administrating a single dose of erythropoietin has better effects on IRI (which could be due to its fewer side effects) than administration of multiple doses.

In another study we reported that erythropoietin pre-treatment for 48 and 96 hours were associated with a significant increase in hemoglobin, hematocrit and brain edema that may explain the adverse consequences due to the side effects (Bigdeli, Mostafavi, Gholamzadeh, & Eskandari, 2016). In addition, a moderate hyperglycemia was observed in 40% of individuals without diabetes in the acute phase of stroke (Quinn, Dawson, & Walters, 2011). Hyperglycemic and normoglycemic patients show more severe neurological defects and poor clinical outcomes (Bhalla et al., 2001; Dietrich, Alonso, & Busto, 1993; Radermecker & Scheen, 2010).

Our research demonstrated that erythropoietin increased blood glucose levels in all groups, especially in the acute phase of stroke. The increase in blood glucose was approximately dose-dependent and higher blood glucose values were correlated with poor clinical and histological outcomes. However unlike the other studies in diabetic rats, erythropoietin increased blood sugar. Several studies have reported a possible link between erythropoietin level and hypoglycemia (Chen et al., 2015).

Hypoglycemia exacerbates stress responses. Stress response causes a further increase in blood glucose in hypoglycemia, compared to normoglycemia condition (Ceriello et al., 2012). This may suggest that erythropoietin pretreatment decreases blood glucose in non-diabetic rats before stroke induction and exacerbates hyperglycemia resulting from stress response after IRI. The lack of weight gain among the treated animals with higher doses of erythropoietin confirms this hypothesis.

Decreased glucose levels following the treatment with erythropoietin in rats with a high-fat diet for 2 weeks was associated with weight loss in them (Meng et al., 2013). Also, decreasing blood glucose over a period of 24 hours in all groups can be explained by stress response. In addition, previous studies reported a connection between blood glucose levels, inflammatory markers and the activation of stress response after stroke (Meng et al., 2013; Shukla et al., 2017). Additionally, several studies have demonstrated that ischemia-reperfusion is associated with enhanced inflammatory factors such as IL-1, IL-1β, TNF-α (tumor necrosis factor-alpha), and IL-6. It is also correlated with worse histological outcomes in which erythropoietin pre-treatment protected tissue from IRI by reducing these factors (Liao et al., 2016; Liu et al., 2015; Nandra et al., 2013; Rong & Xijun, 2015; Wu et al., 2006).

In connection with inflammatory markers, we discovered that a single dose of erythropoietin pretreatment decreased levels of IL-1α and IL-6, compared to the control group. On the other hand, the groups with higher levels of inflammatory markers showed worse tissue results. Moreover, our results identified an association between inflammatory markers and blood glucose level. Meng et al. (2013) study on high-fat diet-fed mice along with the increment of blood glucose, reported an increased in IL-6 and TNF-α. Based on previous studies, the main determinants of blood glucose concentration were cortisol, glucagon and insulin (O’neill, Davies, Fullerton, & Bennett, 1991).

Ramirez reported that the treatment of anemia in dialysis patients with recombinant human erythropoietin hormone could affect the pituitary-hypothalamic-adrenal axis and increase responses of adrenocorticotropin hormone to corticotropin-releasing hormone. However, to confirm the hypothesis of hyperglycemia after hypoglycemia and stress response occurrence in brain ischemia following erythropoietin pretreatment, it is necessary to measure blood glucose before stroke induction and cortisol level in the early hours after the stroke. Evaluating the above-mentioned parameters is recommended for further studies (Ramirez, Bittle, Sanders, Rabb, & Bercu, 1994).

## 5. Conclusion

In conclusion, our results suggest an association between hyperglycemia, levels of inflammatory factors and severity of injury after the IRI by MCAO model in rats. In addition, erythropoietin pretreatment de-creased IRI by reducing inflammatory factors, establishing stable physiological conditions, as well as improving infarct volume and neurological deficits. It appears that its effects are dose-dependent. Our results recommend that the administration of higher erythropoietin doses should be taken with caution due to its possible side effects. However, further investigations are required to determine more detailed effects of erythropoietin on physiological conditions and stress response over activation after IRI in the brain.

## Ethical Considerations

### Compliance with ethical guidelines

All animal experiments have been conducted in accordance with the International Standard for Animal Ethics, under the supervision of the Ethics Committee of Zanjan University of Medical Sciences.
